# A Consistency Model for Identifying the Effects of n-3 and n-6 Fatty Acids on Lipoproteins in Dialysis Patients

**DOI:** 10.3390/nu14061250

**Published:** 2022-03-16

**Authors:** Ke-Yu Chang, Yi-Chun Chen, Shu-Ching Yeh, Chih-Chin Kao, Chung-Yi Cheng, Yi-No Kang, Chih-Wei Huang

**Affiliations:** 1 Department of General Medicine, Taipei Medical University Hospital, Taipei 110, Taiwan; b101100068@tmu.edu.tw; 2 Department of Emergency Medicine, Taipei Medical University Hospital, Taipei 110, Taiwan; mow777gj@yahoo.com.tw; 3 Division of Nephrology, Department of Internal Medicine, Taipei Medical University Hospital, Taipei 110, Taiwan; dr.yeh123@gmail.com (S.-C.Y.); 121008@h.tmu.edu.tw (C.-C.K.); 4 Division of Nephrology, Department of Internal Medicine, School of Medicine, College of Medicine, Taipei Medical University, Taipei 110, Taiwan; 94426@w.tmu.edu.tw; 5 Division of Nephrology, Department of Internal Medicine, Wan Fang Hospital, Taipei Medical University, Taipei 116, Taiwan; 6 TMU Research Center of Urology and Kidney (TMU-RCUK), Taipei 110, Taiwan; 7 Evidence-Based Medicine Center, Wan Fang Hospital, Taipei Medical University, Taipei 116, Taiwan; academicnono@gmail.com; 8 Research Center of Big Data and Meta-analysis, Wan Fang Hospital, Taipei Medical University, Taipei 116, Taiwan; 9 Cochrane Taiwan, Taipei Medical University, Taipei 110, Taiwan; 10 Institute of Health Policy and Management, College of Public Health, National Taiwan University, Taipei 100, Taiwan; 11 Department of Health Care Management, College of Health Technology, National Taipei University of Nursing Health Sciences, Taipei 112, Taiwan; 12 International Center for Health Information Technology, College of Medical Science and Technology, Taipei Medical University, Taipei 106, Taiwan

**Keywords:** dialysis, n-3, n-6, low-density lipoprotein, high-density lipoprotein

## Abstract

Numerous randomized controlled trials (RCTs) and meta-analyses have assessed the effects of supplemental dietary polyunsaturated fatty acids (PUFAs) on levels of low-density lipoprotein (LDL) and high-density lipoprotein (HDL) and the LDL/HDL ratio in patients receiving renal replacement therapy (RRT). However, results are ambiguous due to mixed reports of various nutrients used in the intervention group. We performed a network meta-analysis of RCTs to assess the effects of PUFAs on lipid profiles in patients undergoing RRT. RCTs performed before November 2021 were gathered from three databases. The means, standard deviations and the number of cases for each arm were independently extracted by two authors to form a network meta-analysis of LDL and HDL levels and the LDL/HDL ratio in a random effects model. Twenty-eight RCTs (*n* = 2017 subjects) were included in this study. The pooled results revealed that the combination of omega-3 fatty acids (n-3) and omega-6 fatty acids (n-6) produced significantly lower LDL (standardized mean difference (SMD) = −1.43, 95% confidence interval: −2.28 to −0.57) than the placebo. Both n-3 fatty acids (SMD = 0.78) and the combination of n-3 + n-6 (SMD = 1.09) benefited HDL significantly compared with placebo. Moreover, n-3 alone also exhibited a significantly lower LDL/HDL ratio than placebo. Collectively, PUFAs seem to be adequate nutrients for controlling lipoproteins in patients undergoing RRT. Specifically, n-3 + n-6 supplementation improved LDL levels, while n-3 improved HDL levels and the LDL/HDL ratio. However, our data provide limited information on specific dosages of PUFAs to form a concrete recommendation.

## 1. Introduction

Patients with chronic kidney disease (CKD) and dialysis suffer from multiple complications, including anemia, protein loss, uremic bleeding, secondary hyperparathyroidism and cardiovascular diseases (CVDs). CVDs are the most common cause of morbidity and mortality in patients with end-stage renal disease undergoing hemodialysis [1,2]. The accumulation of cholesterol in the endothelium of blood vessels is the usual cause of atherosclerosis, which can progress to CVD [3]. Although lipid-modifying drugs are prescribed to prevent the development of CVD in patients with CKD [4], some studies have shown controversial results regarding the efficacy of statins in dialysis patients [5,6]. The benefits of therapeutic strategies other than pharmacological interventions, such as dietary supplements, should be evaluated for efficacy.

Polyunsaturated fatty acids (FAs; PUFAs), a class of dietary supplements with more than two double bonds, the first of which can be located three, six, or nine carbons away from the omega end, have shown little or no effect in preventing CVDs in the general population [7]. However, numerous studies have assessed the effects of PUFAs on lipid profiles in dialysis patients. Randomized controlled trials (RCTs) have yielded controversial effects of PUFAs on the levels of low-density lipoprotein (LDL)-cholesterol (C) and high-density lipoprotein (HDL)-C in dialysis patients [8,9,10,11,12,13,14,15,16,17,18,19,20,21,22,23,24,25,26,27,28,29,30,31,32,33,34,35,36,37]. Interestingly, previous meta-analyses of RCTs showed that PUFAs have beneficial effects in treating dialysis patients [38,39]. One meta-analysis supported omega-3 FAs significantly lowering serum triglycerides (TGs) and LDL-C [38]. Another meta-analysis, the largest systematic review available, showed that PUFAs decrease LDL levels and increase HDL levels [39]. However, the study’s findings on LDL (*I*^2^ = 78.3%) and HDL (*I*^2^ = 91.5%) were highly heterogeneous, most likely due to the diversity of the interventions. Although that study attempted to explore sources of heterogeneity by stratifying data according to characteristics, the heterogeneity could not successfully be reduced. Further detailed analyses of such interventions would improve the quality of the pooled results and provide a better understanding of PUFAs’ effects in dialysis patients [40]. In our experience, different regimens should also be evaluated more precisely. For instance, many studies frequently used vitamin E, a fat-soluble antioxidant, in the control or placebo group [9,41,42,43]. Thus, we consider it essential to distinguish the cause of the effects on lipoprotein in people undergoing dialysis.

In addition, these previous meta-analyses usually focused on LDL, HDL, or total cholesterol (TC) in assessing lipid profiles and CVDs. With evidence indicating that the LDL/HDL ratio is also an essential index for predicting CVDs [44,45], an improved meta-analysis study design could provide more robust conclusions about the effects of PUFAs on lipoprotein levels in dialysis patients. Therefore, the purpose of our study was to clarify whether using PUFAs can improve lipid profiles (LDL, HDL and the LDL/HDL ratio) in dialysis patients through a systematic review with a network meta-analysis of RCTs.

## 2. Methods

In this advanced review work, team members comprised nephrologists and experienced researchers in network meta-analysis (CRD42020150743). Our methods in this systematic review followed the Cochrane handbook and the reporting of this study followed PRISMA guidance [46]. The data we used in this study were published in previous RCTs. Therefore, this work allows for the exemption from institutional review board approval. Based on our study purpose, we formed a PICO (persons, interventions, controls and outcomes) question:

P: Patients receiving dialysis including both hemodialysis and peritoneal dialysis;

I: PUFA treatments;

C: Placebo or non-PUFA treatments; and

O: LDL and HDL levels and the LDL/HDL ratio.

### 2.1. Study Selection Criteria

According to our PICO question, we defined the eligibility criteria for evidence selection and inclusion criteria as follows: (a) patients undergoing dialysis; (b) patients treated with omega-3 FAs, omega-6 FAs, or combination treatment with PUFAs; and (c) patients randomly allocated into two or more groups. This comprehensive review removed studies if: (a) the articles were gray literature without detailed information about the trial design, such as conference or meeting abstracts which were not formally published in traditional or commercial publishing (https://training.cochrane.org/handbook/current/chapter-04#section-4-3-5 (accessed on 30 January 2022)); (b) the study did not separate dialysis patients from non-dialysis patients when it concurrently recruited both populations; and (c) the article did not report LDL, HDL, or the LDL/HDL ratio after treatment.

### 2.2. Search Strategy and Study Selection

Our data were mainly from three important biomedical databases: Embase, PubMed and Web of Science. The primary search strategy used relevant terms of dialysis and PUFAs in PubMed. The terms we used involved natural texts and medical subject headings. Our search applied relevant MeSH terms in PubMed and Emtree in Embase. We combined relevant terms of dialysis using the Boolean operator “OR” and also used Boolean operator “OR” to combine relevant PUFA terms. Then, we connected the dialysis and PUFA parts using the Boolean operator “AND”. There was no restriction on language or publication date in this search strategy. We adopted this search strategy for Embase and Web of Science. The last search was completed for references before November 2021. Complete information about the systematic search is shown in File S1.

Two researchers independently selected evidence according to the eligibility criteria through two steps. They screened titles and abstracts of those references selected by the systematic search. Then, the researchers excluded ineligible references after a full-text review. Our study team met to make a final judgment of evidence selection if the two researchers exhibited discrepancies in their selections.

### 2.3. Data Extraction and Quality Assessment

The two researchers also independently extracted data and assessed the quality of each trial we selected for our systematic review. They extracted details from the trial design, location, inclusion year, treatments, numbers of patients, mean age, sex, dialysis period, LDL, HDL and the LDL/HDL ratio at the end of treatment. Our team members extracted the mean and standard deviation (SD) because LDL, HDL and the LDL/HDL ratio were usually presented as continuous outcomes. We calculated the SD from the standard error (SE) using SE = SD/√N if the original report only showed the SE. When the original report only gave the interquartile range (IQR), our team members estimated the SD according to the formula IQR/1.35. If we only found the maximum and minimum, our team estimated the SD based on Hozo’s method [47].

After data extraction, the two researchers assessed the risk of bias in each trial using the Cochrane Risk of Bias Tool. The assessment evaluated randomization generation, allocation concealment, blinding of participants and personnel, selecting reports, incomplete outcome data and other sources of bias. Our study team had another meeting to make a final judgment of the quality assessment if there were any disagreements during the evaluation between the two researchers.

### 2.4. Evidence Synthesis and Statistical Analysis

Our study qualitatively synthesized evidence and appropriately pooled quantitative data through a contrast-based network meta-analysis in a random effects model. The basic concept for frequentist network meta-analysis is to combine a direct effect and indirect effect size. For example, a three-node network meta-analysis could be understood as follows:

ES _(total)_ of A vs. B = ES _(dir.)_ A vs. B + ES _(ind.)_ A vs. B (1)

ES _(ind.)_ A vs. B = ES _(dir.)_ C vs. B − ES _(dir.)_ C vs. A (2)

Because LDL, HDL and the LDL/HDL ratio at the end of treatment were continuous data, our consistency model performed a weighted mean difference (WMD) with the 95% confidence interval (CI). If the original data were in different measurement units, we applied the standardized mean difference (SMD) to resolve the differences in the analysis. For instance, those data could be in mg/L, mg/dL, ng/L, ng/mL, or pg/mL. Inconsistency could be tested by the concept as follows:

Inconsistency of A vs. B = ES _(ind.)_ A vs. B − ES _(dir.)_ A vs. B (3)

Var of inconsistency of A vs. B = Var _(dir.)_ A vs. B + Var _(ind.)_ A vs. B (4)

A comparison-adjusted funnel plot and Egger’s test were performed for detecting potential small-study effects. All analyses we mentioned above were conducted in R version 4.1.0 for Microsoft Windows via RStudio version 1.4.1717 (Public Benefit Corporation, Boston, MA, USA) using package ‘netmeta’ (2.0-1) and ‘meta’ (5.1-0).

### 2.5. Confidence Rating for Network Meta-Analysis

After data synthesis, the present study further rated confidence of each outcome by comparison according to within-study bias, reporting bias, indirectness, imprecision, heterogeneity and incoherence. The rating consisted of four levels: high, moderate, low and very low. Within-study bias, indirectness, imprecision, heterogeneity and incoherence were evaluated based on methodological rules and can be judged as no concerns, some concerns, or major concerns. Reporting bias can be considered low risk, some concerns and high risk. Due to the complexity of the consistency model in the present study, a summary of confidence rating was mainly presented for significant findings.

## 3. Results

**Figure 1:**
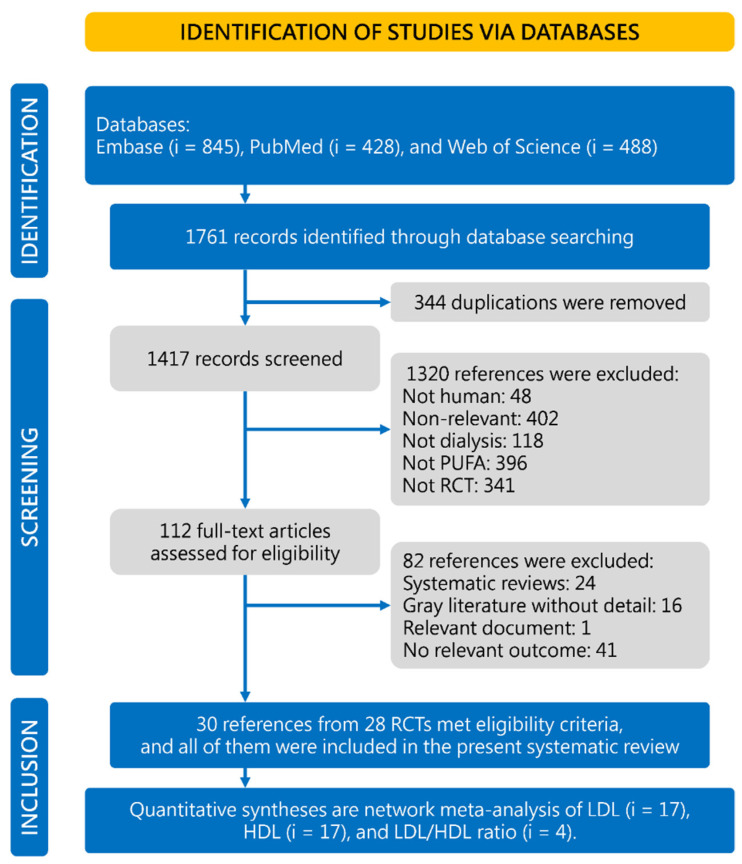
Flowchart of the systematic review and meta-analysis. HDL, high-density lipoprotein; LDL, low-density lipoprotein; PUFA, polyunsaturated fatty acid; RCT, randomized controlled trial.

This study identified 1761 references from Embase (*i* = 845), PubMed (*i* = 428) and Web of Science (*i* = 488). Those duplicates (*i* = 344) and irrelevant references (*i* = 1305) were removed after screening the titles and abstracts. Twenty-four systematic reviews, 41 RCTs without relevant outcomes, 16 articles of gray literature without details and a relevant document (protocol) were excluded from our systematic review. Finally, 30 references from 28 RCTs met the eligibility criteria (Figure 1) [8,9,10,11,12,13,14,15,16,17,18,19,20,21,22,23,24,25,26,27,28,29,30,31,32,33,34,35,36,37,48].

### 3.1. Characteristics and Quality of the Included Studies

The 28 RCTs recruited 2017 dialysis patients from Brazil [13,24], Canada [14], Denmark [15,30,32,34,35,36], Egypt [10], Iran [9,16,17,18,19,21,22,25,26,27,28,29,37], Korea [8,23], Spain [48] and the United States [11,12,31]. These trials could be categorized into eight treatment strategies, including placebo, vitamin E and medium-chain triglycerides (MCTs), omega-3 FAs, the combination of omega-3 FAs and vitamin E (n-3 + VitE), the combination of omega-6 FAs and vitamin E (n-6 + VitE), the combination of n-3 + n-6 and combination of n-3 + n-6 + VitE. Table 1 presents relevant information about the trial location, inclusion years, treatments, mean age, sex and dialysis period. File S2 presents the results of the quality assessment of the included trials.

### 3.2. Low-Density Lipoprotein (LDL)

**Figure 2:**
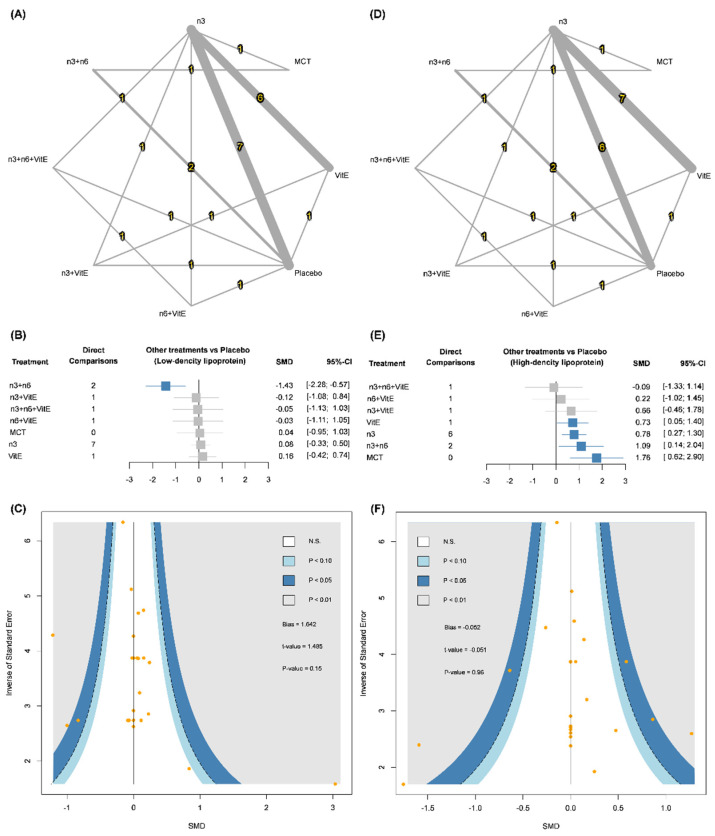
(**A**) Network plots of low-density lipoprotein, (**B**) forest plot of low-density lipoprotein, (**C**) funnel plot of low-density lipoprotein, (**D**) network plots of high-density lipoprotein, (**E**) forest plot of high-density lipoprotein and (**F**) funnel plot of high-density lipoprotein. CI, confidence interval; MCTs, medium chain triglycerides; n3, omega-3 fatty acids; n6, omega-6 fatty acids; SMD, standardized mean difference; Vit E, vitamin E. Results in the consistency model without direct comparison can be estimated by Bucher’s adjusted indirect comparison method.

**Figure 3:**
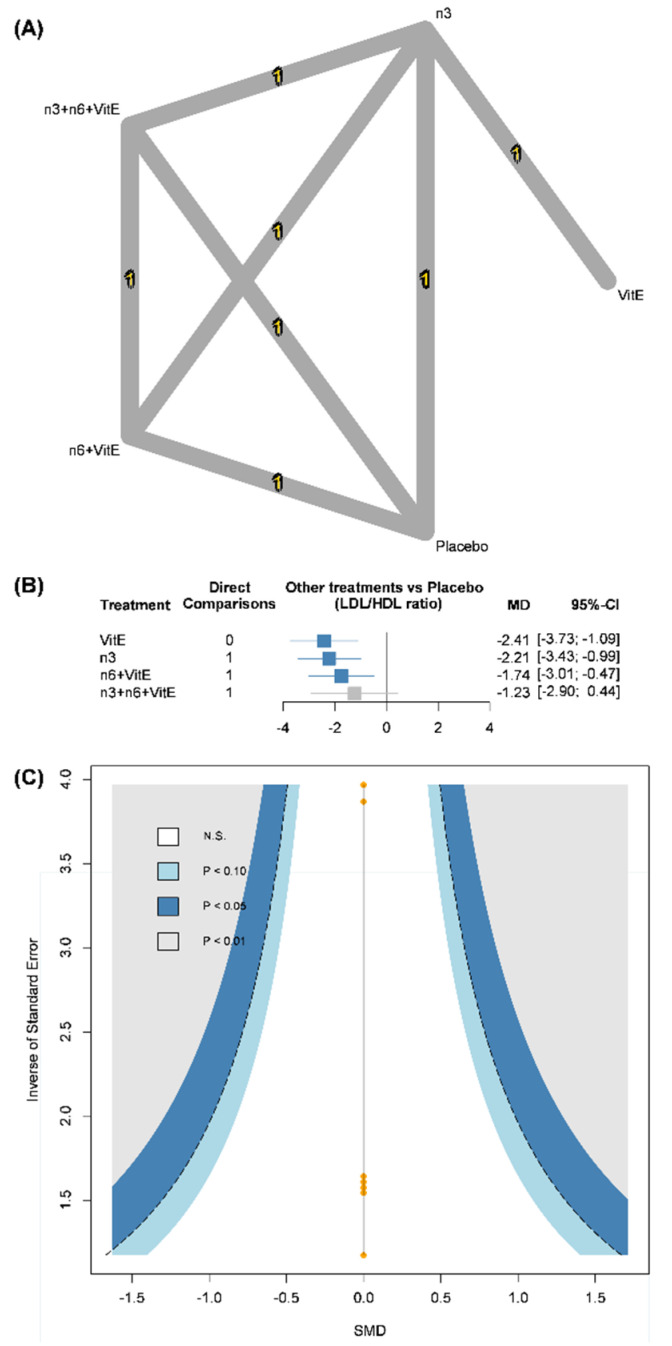
(**A**) Network plots of low-density lipoprotein to high-density lipoprotein ratio, (**B**) forest plot of low-density lipoprotein to high-density lipoprotein ratio, (**C**) comparison-adjusted funnel plot of low-density lipoprotein to high-density lipoprotein ratio. CI, confidence interval; MCTs, medium chain triglycerides; n3, omega-3 fatty acids; n6, omega-6 fatty acids; MD, mean difference; Vit E, vitamin E.

In total, 17 RCTs with 1059 dialysis patients receiving omega-3 FAs, n-3 + VitE, n-6 + VitE, n-3 + n-6, n-3 + n-6 + VitE, vitamin E, MCTs and placebo were included in the network meta-analysis of LDL (Figure 2A) [9,13,14,15,19,20,21,22,23,25,26,30,32,33,34,35,36,37,48]. The pooled estimate showed that with placebo as the reference, n-3 + n-6 was the only nutritional supplement that achieved significantly lower LDL (SMD: −1.43, 95% CI: −2.28 to −0.57; Figure 2B) and n-3 + n-6 also showed significantly lower LDL than other nutritional supplements (Table 2). As to the quality of the pooled estimate for LDL, a design-by-treatment interaction model and Egger’s test did not reveal significant inconsistencies (Q statistics = 0.49; *p* > 0.05; File S3) or publication bias (point estimate = 1.642; *p* > 0.05; Figure 2C) in the network meta-analysis.

### 3.3. High-Density Lipoprotein (HDL)

Seventeen RCTs with 998 dialysis patients formed an eight-node network meta-analysis for HDL (Figure 2D) [9,13,15,19,20,21,22,23,25,26,28,30,32,33,34,35,36,37,48]. The pooled estimate demonstrated that with placebo as the reference, omega-3 FAs (SMD: 0.78, 95% CI: 0.27 to 1.30), n-3 + n-6 (SMD: 1.09, 95% CI: 0.14 to 2.04), vitamin E (SMD: 0.73, 95% CI: 0.05 to 1.40) and MCTs (SMD: 1.76, 95% CI: 0.62 to 2.90) exhibited significantly higher HDL (Figure 2E). The design-by-treatment interaction model detected global inconsistencies among the included RCTs (Q statistics = 20.68; *p* < 0.05) and further design-specific decomposition within the designs showed potential threats in the comparison of placebo and n-3 + n-6 (Q statistics = 18.50; *p* < 0.05; File S4), while mean path length of the comparison did not reach critical threshold. The biggest concern among the significant findings was that mean path length between placebo and MCT passed the critical threshold (File S4). The Egger’s test showed no significant publication bias (point estimate = −0.052; *p* > 0.05) in the network meta-analysis of HDL (Figure 2F).

### 3.4. Low-Density Lipoprotein/High-Density Lipoprotein (LDL/HDL) Ratio

Available data of the LDL/HDL ratio from the included trials formed a five-node network meta-analysis (omega-3 FAs vs. n-6 + VitE vs. n-3 + n-6 + VitE vs. vitamin E vs. placebo). However, few trials provided data based on 114 cases (Figure 3A) [12,19]. The pooled result showed that the LDL/HDL ratio in omega-3 FA (WMD: −2.21, 95% CI: −3.43 to −0.99), n-6 + VitE (WMD: −1.73, 95% CI: −3.01 to −0.47) and vitamin E (WMD: −2.41, 95% CI: −3.73 to −1.09) groups were significantly lower than those in the placebo group. Interestingly, n-3 + n-6 + VitE only had a limited benefit on the LDL/HDL ratio with no statistical significance compared to the placebo (Figure 3B). Due to this, the consistency model included a four-arm trial and the design-by-treatment interaction model could not be performed. In the meantime, all mean path lengths in the consistency model did not reach the critical threshold (File S5). Egger’s test could not be performed due to limited evidence, but the comparison-adjusted funnel plot was not asymmetric (Figure 3C).

## 4. Discussion

Our study provides the first evidence from a network meta-analysis investigating the effects of PUFAs on lipid profiles in dialysis patients. This study synthesized 28 RCTs involving 2017 patients undergoing dialysis and formed a network of placebos, vitamin E, MCT, omega-3 FAs, n-3 + VitE, n-6 + VitE, n-3 + n-6 and n-3 + n-6 + VitE. We examined three critical predictors of CVDs: LDL, HDL and the LDL/HDL ratio. The networks for LDL and HDL consisted of eight treatments and the network for the LDL/HDL ratio consisted of placebo, omega-3 FAs, vitamin E, n-6 + VitE and n-3 + n-6 + VitE. We observed that PUFAs might benefit LDL, HDL and the LDL/HDL ratio. Unfortunately, confidence in these findings were low and very low because of within-study bias and heterogeneity (Table 3).

In contrast to previous findings, our meta-analysis study showed that omega-3 FAs and MCTs were beneficial in improving HDL levels in dialysis patients. Previous meta-analyses considered all placebo groups equally and concluded that omega-3 FAs had no effects on serum HDL [38,39]. In contrast, our study separated MCTs and omega-6 FAs from pure placebos. We found that RCTs reporting no omega-3 FAs on HDL had the same problem by including MCTs in the placebo group [22,25,26]. In contrast, RCTs not using MCTs as placebos tended to show significant benefits of omega-3 FAs on increasing serum HDL, which was also consistent with our study [15,19]. Previous studies stated that MCTs were associated with increased serum HDL in the general population and animal models and our meta-analysis further confirmed these benefits for dialysis patients [57,58,59].

As mentioned above, the LDL/HDL ratio was proven to be an independent predictor of CVD and sudden cardiac death, with more potency than serum LDL or HDL alone [44,45]. Moreover, most lifestyle interventions mainly focus on decreasing LDL and increasing HDL; therefore, the LDL/HDL ratio has become a critical biomarker of CVD risk. However, this ratio has seldom been reported in studies investigating the effects of PUFAs on lipid profiles in dialysis patients, leaving a knowledge gap that our study attempted to address. Discussions regarding the effect of vitamin E on LDL/HDL ratio have suggested that increasing oxidative stress fosters dyslipidemia and atherosclerosis in dialyzed patients [60,61,62], with vitamin E in both oral medication and dialyzer forms being able to effectively modify lipid profiles through a reduction in oxidative stress [63,64]. Our findings, in agreement with these previous studies, showed convincingly that omega-3 FAs and vitamin E significantly decreased the LDL/HDL ratio compared to placebo. However, the effects of PUFAs on the LDL/HDL ratio appear to be weakened if they and vitamin E are administered simultaneously. This might be due to reducing the peroxidation of PUFAs by vitamin E.

Although some recent meta-analyses studied the effects of PUFAs on the lipid profile, many outcomes showed high heterogeneity [38,39]. We considered that the limitations of those studies lay in not exploring differences among regimens, especially regimens using placebo. In our opinion, supplementation with PUFAs, vitamin E and MCTs helps modify lipid profiles by controlling the inflammatory status in patients undergoing dialysis. Due to dialysis patients undergoing a reduction in renal excretion and increasing polypharmacy and comorbidity, which results in statins, use for these patients might not be as effective as in general populations [5,6]. Another good meta-analysis in 2019 indicated that omega-3 FAs lower cardiovascular mortality, although the systematic review did not analyze lipid profiles [65]. Our evidence provides a possible explanation for their finding and fosters an understanding of the effects of PUFAs on cardiovascular outcomes through affecting the lipid profiles of dialysis patients.

### Limitations

Our systematic review has three limitations. First, many dialysis patients have CVD. Unfortunately, the baseline CVD characteristics were not reported using the same standards. Second, the results of taking PUFA supplements are still unclear, even though we separated n-3 + n-6 and n-3 + VitE from n-3. Determining the effectiveness of different supplement combinations and should improve our understanding PUFAs’ therapeutic use, though our evidence cannot yet guide specific dosages in dialysis cases. Third, data in the present consistency model are short-term findings (within three months) and the long-term effects of PUFAs supplements need to be subjected to further study. Fourth, network meta-analysis of the LDL/HDL ratio was based on limited evidence and thus, the pooled estimate may be underpowered. In addition, we found no available data for n-3 + VitE. Therefore, we suggest that future studies present the LDL/HDL ratio to show whether PUFAs improve lipid profiles, especially among dialysis patients.

## 5. Conclusions

Based on an advanced method in our study, PUFA supplementation could be an option for controlling lipid profiles in dialysis patients. n-3 + n-6 PUFAs decrease LDL levels and increase HDL levels. As well, even without n-6, n-3 FAs increased HDL levels and reduced the LDL/HDL ratio. However, our results offer limited information about PUFA dosages for guiding the clinical treatment of dialysis patients. Therefore, we suggest that further studies should design multiple arms to clarify relationships among PUFA dosages and improved lipoprotein levels and ratios in patients undergoing dialysis.

## Figures and Tables

**Table 1:** Characteristics of the included randomized controlled trials.

Lead Author	Location	Inclusion Year	Treatments	No. of Patients	Mean Age (Years)	Sex (M/F)	Dialysis Period (Years)	Relevant Outcome
An	Korea	2010	1. n-3	23 ^a^	56.7	12/11	3.42	HDL ^d^ and
[8]			2. Placebo	20 ^a^	58.1	8/12	4.31	LDL ^d^
Asemi	Iran	2014	1. n-3	30 ^b^	55.2	20/10	3.6	HDL and
[9]			2. Vit E ^(αT)^	30 ^b^	61.2	20/10	3.5	LDL
			3. n-3 + Vit E ^(αT)^	30 ^b^	54.9	20/10	3.4	
			4. Placebo	30 ^b^	59.9	20/10	3.4	
Ateya	Egypt	2015	1. n-3	25 ^b^	14.7	14/11	NR	HDL ^d^ and
[10]			2. Vit E	24 ^b^	14.6	13/11	NR	LDL ^d^
Bowden	USA	NR	1. n-3	44 ^b^	59.3	25/19	1.83	HDL ^d^ and
[11]			2. n-3 + Vit E ^(Corn oil)^	43 ^b^	60.8	20/23	2.39	LDL ^d^
Daud	USA	NR	1. n-3	31 ^b^	59	20/11	3.6	LDL/HDL
[12]			2. Vit E ^(Olive oil)^	32 ^b^	58	12/20	3.3	
de Mattos	Brazil	2012 to	1. n-3	43 ^b^	52.7	27/16	5.49	HDL and
[13]		2013	2. Placebo ^(Soybean oil)^	45 ^b^	51.3	30/15	3.63	LDL
Donnely	Canada	NR	1. n-3 + Vit E	16 ^a^	51	12/4	NR	HDL
[14]			2. Vit E ^(Olive oil)^	16 ^a^	51	12/4	NR	
Ewers	Den-	2007	1. n-3	40 ^b^	64.6	30/10	NR	HDL and
[15]	mark		2. Placebo ^(NS)^	40 ^b^	64.6	30/10	NR	LDL
Gharekhani	Iran	NR	1. n-3	25 ^b^	56.8	12/13	5	HDL ^d^ and
[16]			2. Placebo ^(Paraffin)^	20 ^b^	57.2	8/12	6	LDL ^d^
Jabbari	Iran	NR	1. n-3	57 ^b^	64.58	42/15	24	HDL ^e^ and
[17]			2. Placebo	60 ^b^	61.05	33/27	12	LDL ^e^
Kajbaf	Iran	NR	1. n-3	26 ^b^	57.76	17/9	NR	HDL ^d^ and
[18]			2. Placebo	26 ^b^	58.34	19/7	NR	LDL ^d^
Khajehdeh	Iran	NR	1. n-3	15 ^b^	32.7	8/7	2.19	HDL,
[19]			2. n-3 + Vit E ^(Corn oil)^	15 ^b^	33.6	8/7	2.46	LDL, and
			3. n-3 + n-6 ^(Sesame oil)^	15 ^b^	32.3	8/7	2.83	LDL/HDL
			4. Placebo	15 ^b^	31.1	7/8	2.21	
Khalatbari	Malaysia	NR	1. n-3 + n-6 ^(flaxseed)^	15 ^b^	54	10/5	2.58	HDL and
[20]			2. Placebo	15 ^b^	54.5	6/9	2.83	LDL
Khorsro-	Iran	NR	1. n-3	44 ^b^	51.5	32/12	NR	HDL and
shahi [21]			2. Placebo ^(Soft pill)^	44 ^b^	48.6	31/13	NR	LDL
Kooshki	Iran	NR	1. n-3	17 ^b^	50	10/7	1.75	HDL and
[22]			2. Placebo	17 ^b^	50	11/6	2.3	LDL
Lee	Korea	2012	1. n-3	8 ^b^	60	2/6	NR	HDL and
[23]			2. Vit E ^(Olive oil)^	7 ^b^	64.4	3/4	NR	LDL
Lemos	Brazil	NR	1. n-3 + n-6 + Vit E ^(flaxseed oil and αT)^	70 ^b^	55.7	39/31	2.4	HDL ^d^ and
[24]			2. Placebo + Vit E ^(Mineral oil and αT)^	75 ^b^	58.3	46/29	2.9	LDL ^d^
Mirfatahi	Iran	2014 to	1. n-3 + n-6 ^(flaxseed oil)^	17 ^b^	68	12/5	4.4	HDL and
[25]		2015	2. MCTs	17 ^b^	59	10/7	4.6	LDL
Moeinzadeh	Iran	NR	1. n-3	26 ^b^	57.76	17/9	NR	HDL ^d^ and
[27]			2. Placebo	26 ^b^	58.34	19/7	NR	LDL ^d^
Naini	Iran	2012	1. n-3	45 ^c^	57.7	24/21	NR	LDL
[28]			2. Placebo	45 ^c^	59.3	27/18	NR	
Omrani	Iran	2013 to	1. n-3	29 ^b^	55	16/13	NR	HDL ^f^ and
[29]		2014	2. Vit E	29 ^b^	56	19/10	NR	LDL ^f^
Rantanen	Den-	2014 to	1. n-3	56 ^a^	64.2	37/19	1	HDL and
[30]	mark	2016	2. Vit E ^(Olive oil)^	56 ^a^	60.5	37/19	2.2	LDL
Ruperto	Spain	2018	1. n-3	21 ^b^	66	16/5	Overall:	HDL and
			2. Placebo	21 ^b^	68	13/8	7.5	LDL
Saifullah	USA	2006	1. n-3 + n-6 + Vit E	15 ^b^	58	11/4	NR	HDL ^g^ and
[31]			2. n-3 + n-6 + Vit E	8 ^b^	57	7/1	NR	LDL ^g^
Sorensen	Den-	NR	1. n-3	81 ^b^	66	53/28	3	HDL and
[32]	mark		2. Vit E ^(Olive oil)^	80 ^b^	68	51/29	2.17	LDL
Svensson	Den-	NR	1. n-3	28 ^b^	60	16/12	NR	HDL and
[35]	mark		2. Vit E ^(Olive oil)^	30 ^b^	58	23/7	NR	LDL
Svensson	Den-	2002 to	1. n-3	103 ^b^	66	69/34	3.7	HDL and
[36]	mark	2003	2. Vit E ^(Olive oil)^	103 ^b^	68	54/39	3.7	LDL
Taziki	Iran	NR	1. n-3	15 ^b^	47	5/10	3	HDL and
[37]			2. Placebo ^(NS)^	18 ^b^	59.5	6/12	3.5	LDL

^a^, any type of dialysis; ^b^, hemodialysis only; ^c^, peritoneal dialysis only; ^d^, no data on specified time point in this study; ^e^, no data on the end of point but change score only; ^f^, incomplete data report; ^g^, having similar formula in both groups but various doses; F, female; M, male; MCTs, medium-chain triglycerides; n-3, omega-3 fatty acids; n-6, omega-6 fatty acids; NR, no report; NS, no supplement; O, olive oil; P, paraffin; Vit E, vitamin E; αT, alpha-tocopherol.

**Table 2:** League table of low-density lipoprotein and high-density lipoprotein (standardized mean difference with 95% confidence interval).

**Low-Density Lipoprotein**							
MCT							
−0.04 (−1.01; 0.92)	n-3						
*1.47 (0.48; 2.45)*	*1.51 (0.61; 2.42)*	n-3 + n-6					
0.09 (−1.34; 1.52)	0.13 (−0.95; 1.21)	−*1.38 (*−*2.74; −0.01)*	n-3 + n-6 + VitE				
0.16 (−1.17; 1.49)	0.20 (−0.74; 1.14)	−*1.31 (*−*2.58; −0.04)*	0.07 (−1.34; 1.48)	n-3 + VitE			
0.07 (−1.36; 1.50)	0.12 (−0.96; 1.20)	−*1.39 (*−*2.76; −0.03)*	−0.02 (−1.24; 1.21)	−0.08 (−1.49; 1.32)	n-6 + VitE		
0.04 (−0.95; 1.03)	0.08 (−0.33; 0.50)	−*1.43 (*−*2.28; −0.57)*	−0.05 (−1.13; 1.03)	−0.12 (−1.08; 0.84)	−0.03 (−1.11; 1.05)	Placebo	
−0.12 (−1.18; 0.94)	−0.08 (−0.53; 0.38)	−*1.59 (*−*2.59;* −*0.59)*	−0.21 (−1.37; 0.96)	−0.28 (−1.24; 0.69)	−0.19 (−1.36; 0.97)	−0.16 (−0.74; 0.42)	Vitamin E
**High-Density Lipoprotein**							
MCT							
0.98 (−0.13; 2.09)	n-3						
0.67 (−0.46; 1.80)	−0.31 (−1.34; 0.72)	n-3 + n-6					
1.85 (0.22; 3.48)	0.87 (−0.36; 2.11)	1.18 (−0.36; 2.72)	n-3 + n-6 + VitE				
1.10 (−0.43; 2.63)	0.12 (−0.96; 1.21)	0.43 (−1.01; 1.88)	−0.75 (−2.37; 0.87)	n-3 + VitE			
1.54 (−0.08; 3.17)	0.57 (−0.67; 1.80)	0.87 (−0.66; 2.41)	−0.31 (−1.70; 1.09)	0.44 (−1.17; 2.06)	n-6 + VitE		
*1.76 (0.62; 2.90)*	*0.78 (0.27; 1.30)*	*1.09 (0.14; 2.04)*	−0.09 (−1.33; 1.14)	0.66 (−0.46; 1.78)	0.22 (−1.02; 1.45)	Placebo	
1.03 (−0.17; 2.24)	0.05 (−0.44; 0.54)	0.36 (−0.76; 1.48)	−0.82 (−2.14; 0.50)	−0.07 (−1.19; 1.05)	−0.51 (−1.83; 0.81)	*−0.73 (−1.40; −0.05)*	Vitamin E

MCTs, medium chain triglycerides; n-3, omega-3 fatty acids; n-6, omega-6 fatty acids; Vit E, vitamin E.3.3. High-density lipoprotein (HDL). Italics show the significant results of the pairwise comparison analysis.

**Table 3:** Summary of confidence rating of significant findings.

	Within-Study						Confidence
Outcome and Comparison	Bias	Reporting Bias	Indirectness	Imprecision	Heterogeneity	Incoherence	Rating
LDL							
n-3 + n-6 vs. Placebo	Major concerns	Low risk	No concerns	No concerns	Some concerns	No concerns	Very low
n-3 + n-6 vs. MCT	Some concerns	Low risk	No concerns	No concerns	Some concerns	No concerns	Low
n-3 + n-6 vs. VitE	Some concerns	Low risk	No concerns	No concerns	Some concerns	No concerns	Low
n-3 + n-6 vs. n-3	Some concerns	Low risk	No concerns	No concerns	Some concerns	No concerns	Low
n-3 + n-6 vs. n-3 + VitE	Some concerns	Low risk	No concerns	Some concerns	No concerns	No concerns	Low
n-3 + n-6 vs. n-6 + VitE	Major concerns	Low risk	No concerns	Some concerns	No concerns	No concerns	Very low
n-3 + n-6 vs. n-3 + n-6 + VitE	Major concerns	Low risk	No concerns	Some concerns	No concerns	No concerns	Very low
HDL							
MCT vs. Placebo	Some concerns	Low risk	No concerns	No concerns	Some concerns	Major concerns	Very low
VitE vs. Placebo	Some concerns	Low risk	No concerns	No concerns	Some concerns	No concerns	Low
n-3 vs. Placebo	Some concerns	Low risk	No concerns	No concerns	Some concerns	No concerns	Low
n-3 + n-6 vs. Placebo	Major concerns	Low risk	No concerns	No concerns	Some concerns	Major concerns	Very low
LDL/HDL ratio							
Vitamin E vs. Placebo	Major concerns	Low risk	No concerns	N/A ^a^	N/A ^a^	No concerns	Low
n-3 vs. Placebo	Major concerns	Low risk	No concerns	N/A ^a^	N/A ^a^	No concerns	Low
n-6 + VitE vs. Placebo	Major concerns	Low risk	No concerns	N/A ^a^	N/A ^a^	No concerns	Low

^a^ Between study variance cannot be generated for consistency model due to a four-arm randomized controlled trial; MCTs, medium chain triglycerides; N/A, not applicable due to no estimation; n-3, omega-3 fatty acids; n-6, omega-6 fatty acids; Vit E, vitamin E. In our study, n-3 + n-6 significantly lowered LDL levels compared to placebo. However, n-3 alone or a combination of PUFA and vitamin E did not exhibit a beneficial effect. An increase in the serum LDL level and increases in the proportion of oxidized LDL and small, dense LDL particles contribute to an acceleration of atherosclerosis in such patients [49,50,51,52]. Basically, previous studies suggested that PUFA intake can lower serum LDL in general patients, but studies of dialysis patients are still limited [53,54]. In a study with animal models, n-3 + n-6 significantly lowered serum LDL levels in rats [55]. The present meta-analysis further confirmed that that PUFA intake improves serum LDL level and n-3 + n-6 supplementation exhibits more robust efficacy than n-3 supplementation alone. This result is consistent with a previous study showing that PUFA intake lowered serum LDL more than saturated FAs and carbohydrates [56]. Findings from other previous studies indicating that supplementation with omega-3 FAs significantly reduced serum LDL [38,39] were also suggested by our results, though in our case these results fell slightly below statistical significance. Moreover, we considered that the effects of nutritional supplements would be more vigorous in dialysis patients due to their underlying malnutrition status. We cannot process further analysis in our meta-analysis with limited data for head-to-head comparisons of omega-3 FA versus omega-6 FA groups.

## Data Availability

Data described in the manuscript, the codebook and analytic codes will be made available upon request pending application and approval.

## Supplementary Materials

The following supporting information can be downloaded at: https://www.mdpi.com/article/10.3390/nu14061250/s1, File S1: Search strategy. File S2: Quality assessment. File S3: Inconsistency test for the network meta-analysis of low-density lipoprotein. File S4: Inconsistency test for the network meta-analysis of high-density lipoprotein. File S5: Mean path length in network meta-analysis of the low-density lipoprotein (LDL)/high-density lipoprotein (HDL) ratio.
