# Supplementary material for: A Consistency Model for Identifying the Effects of n-3 and n-6 Fatty Acids on Lipoproteins in Dialysis Patients
Source: Nutrients. 2022 Mar 16;14(6):1250. doi: 10.3390/nu14061250 (PMC8954007; doi:10.3390/nu14061250)

Nutrients

A consistency model for identifying the effects of n-3 and n-6 fatty acids on lipoproteins in dialysis patients

(Supplementary Files)

Table of contents

File S1. Search strategy

File S2. Quality assessment

File S3. Inconsistency test for network meta-analysis of low density lipoprotein

File S4. Inconsistency test for network meta-analysis of high density lipoprotein

File S5. Mean path length in network meta-analysis of LDL/HDL ratio.

File S1
Search strategy

Primary search strategy:

#1. dialysis[ti]
#2. hemodialysis[ti]
#3. #1 AND #2
#4. omega 3
#5. omega3
#6. omega-3 fatty acids
#7. omega-3 fatty acid
#8. omega3 fatty acids
#9. omega3 fatty acid
#10. docosahexaenoic
#11. dha
#12. eicosapentaenoic
#13. epa
#14. polyunsaturated
#15. polyunsaturated fatty acids
#16. polyunsaturated fatty acid
#17. LCPUFA*
#18. PUFA*
#19. ω-3
#20. ω3
#21. n3 fatty acid
#22. n3 fatty acids
#23. n-3 fatty acid
#24. n-3 fatty acids
#25. omega-6 fatty acids
#26. omega-6 fatty acid
#27. omega6 fatty acids
#28. omega6 fatty acid
#29. #4 OR #5 OR #6 OR #7 OR #8 OR #9 OR #10 OR #11 OR #12 OR #13 OR #14 OR #15 OR #16 OR #17 OR #18 OR #19 OR #20 OR #21 OR #22 OR #23 OR #24 OR #25 OR #26 OR #27 OR #28
#30. #3 AND #29

File S2

Quality assessment

Study	1	2	3	4	5	6	7
An	Low	Low	High	High	Low	Low	Low
Asemi	Low	Low	Low	Low	Low	Low	High
Ateya	Unclear	High	Low	Low	Low	Low	High
Bowden	Low	Low	Unclear	Unclear	Low	Low	High
Daud	Unclear	High	Low	Low	Low	Unclear	High
de Mattos	Low	Low	Low	Low	Low	Unclear	High
Donnely	Unclear	High	Low	Low	Unclear	Unclear	High
Ewers	Low	Low	High	Low	Low	High	High
Gharekhani	Low	Low	Low	Low	Low	Unclear	Low
Jabbari	Low	High	High	High	Low	High	Low
Kajbaf	Unclear	High	Low	Low	Low	High	Low
Khajehdehi	Unclear	High	High	High	Low	Unclear	Low
Khalatbari	Unclear	High	High	High	Low	High	Low
Khorsroshahi	Unclear	High	High	High	Low	Low	High
Kooshki	Unclear	High	Low	Low	Low	Low	Low
Lee	Low	Unclear	Unclear	Low	Low	Unclear	Low
Lemos	Unclear	High	Low	Low	Low	High	Low
Mirfatahi	Low	Unclear	Unclear	Low	Low	Unclear	Low
Moeinzadeh	Unclear	High	Low	Low	Low	Low	Low
Naini	Unclear	High	Unclear	Unclear	Low	Low	Low
Omrani	Low	Low	Low	Low	Low	Low	Low
Rantanen	Low	Low	Low	Low	Low	Low	Low
Saifullah	Low	Low	Low	Low	Low	Unclear	Low
Ruperto	Low	Low	Unclear	Unclear	Low	Low	Low
Sorensen	Low	Low	Low	Low	Low	Low	Low
Svensson (2007)	Low	Low	Low	Low	Low	High	Low
Svensson (2008)	Low	Low	Low	Low	Low	High	Low
Taziki	Unclear	High	High	Low	Low	Low	High

1. randomization; 2. allocation concealment; 3. blinding of participant and personnel; 4. blinding to outcome assessment; 5. selecting reports; 6. incomplete outcome data, 7. other source of bias.

File S3

Inconsistency test for network meta-analysis of low density lipoprotein

Q statistic to assess consistency under the assumption of a full design-by-treatment interaction random effects model

	Q	df	p-value	tau.within	tau2.within
Between designs	0.49	4	0.9749	0.5984	0.3581

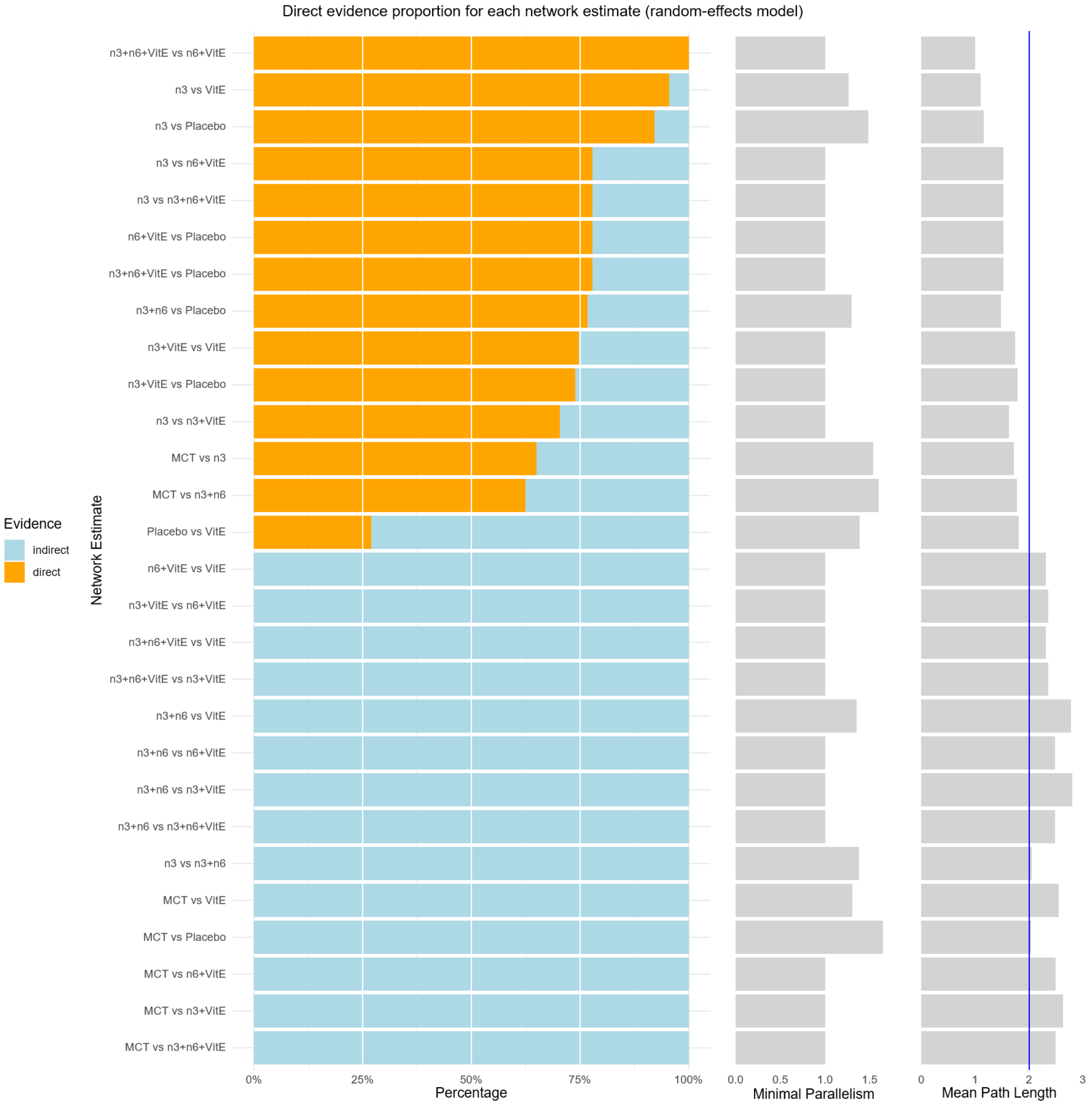

File S4

Inconsistency test for network meta-analysis of high density lipoprotein

Q statistic to assess consistency under the assumption of a full design-by-treatment interaction random effects model

	Q	df	p-value	tau.within	tau2.within
Between designs	20.68	4	0.0004	0.4407	0.1942

Design-specific decomposition of within-designs Q statistic

Design	Q	df	p-value
n3 vs VitE	8.14	5	0.1485
Placebo vs n3	7.43	3	0.0595
Placebo vs n3+n6	18.50	1	< 0.0001

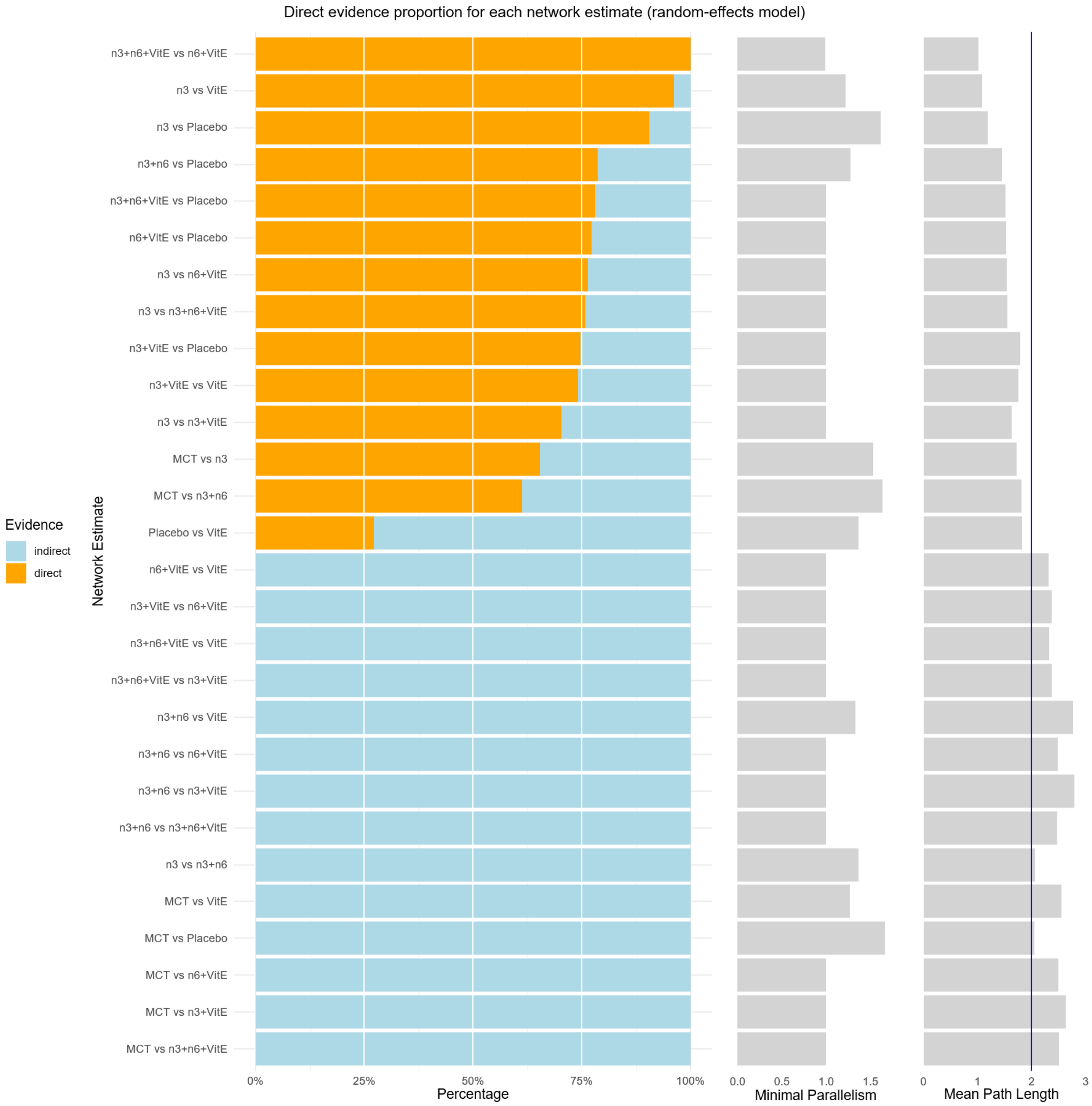

File S5

Mean path length in network meta-analysis of LDL/HDL ratio

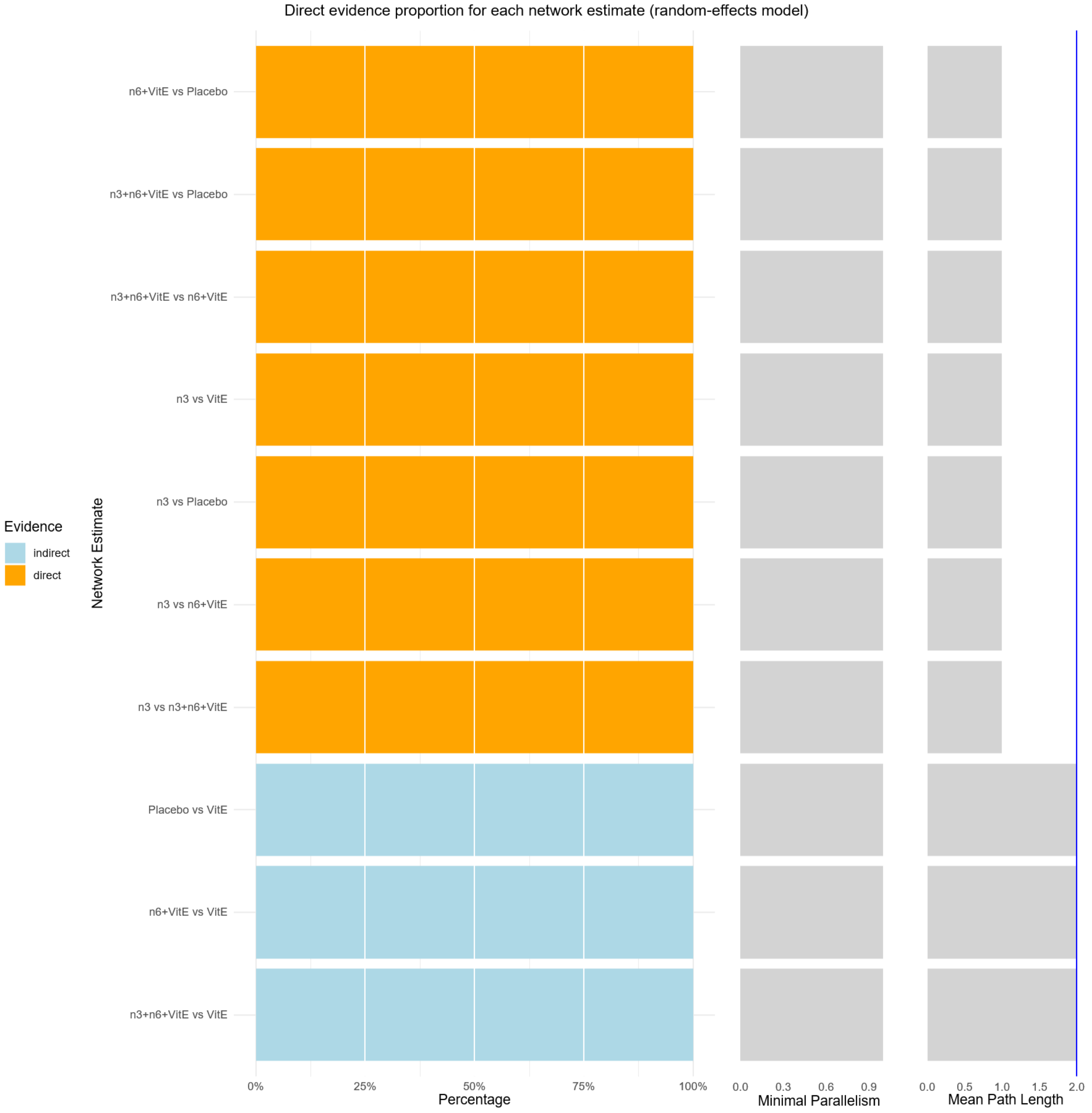

Supplement: Supplementary file 1 [file nutrients-14-01250-s001.zip › nutrients-1606633-supplementary.pdf]
